# Efficacy and Safety of Ramelteon in the Reduction of Delirium and Duration of ICU Stay: A Randomized Placebo-Controlled Trial

**DOI:** 10.7759/cureus.89480

**Published:** 2025-08-06

**Authors:** Akhileshwar, Chakrapani Kumar, Noor Husain, Saajid Hameed

**Affiliations:** 1 Emergency Medicine, Indira Gandhi Institute of Medical Sciences, Patna, IND; 2 Pharmacology, Indira Gandhi Institute of Medical Sciences, Patna, IND

**Keywords:** critical care, delirium, icu, melatonin receptor agonist, ramelteon, sleep quality

## Abstract

Background

Delirium and sleep disturbances are common in critically ill patients and are associated with adverse outcomes, including prolonged intensive care unit (ICU) stays. Ramelteon, a melatonin receptor agonist, may improve sleep and reduce delirium by regulating circadian rhythms. This study evaluated the efficacy of ramelteon in shortening ICU stay, decreasing delirium incidence and duration, and improving sleep quality in critically ill patients.

Methods

A randomized controlled trial was conducted with 60 critically ill patients allocated to either ramelteon (n = 30) or placebo (n = 30). Three participants in the ramelteon group and two in the placebo group were lost to follow-up, leaving 27 and 28 patients for final analysis, respectively. Outcomes included ICU stay duration, delirium frequency and duration, and sleep parameters (total sleep time, awakenings, and nights without awakenings). Statistical analyses were performed using unpaired t-tests and Fisher’s exact test.

Results

The ramelteon group had a significantly shorter mean ICU stay (4.78 vs. 6.23 days, p = 0.0006) and a shorter duration of delirium (18.83 vs. 33.69 hours, p < 0.0001). Delirium incidence was lower in the ramelteon group (7/27, 25.93% vs. 13/28, 46.43%), but this difference was not statistically significant (p = 0.1625). Sleep quality improved significantly, with fewer awakenings per night (0.79 vs. 1.28, p < 0.0001) and more nights without awakenings (0.56 vs. 0.35, p < 0.0001).

Conclusion

Ramelteon was associated with a reduced ICU stay, shorter delirium duration, and improved sleep quality in critically ill patients. While its impact on delirium incidence was not statistically significant, the findings suggest a potential benefit in mitigating delirium severity and enhancing recovery. Further large-scale studies are needed to confirm these results.

## Introduction

Delirium is a common and serious complication experienced by many patients during their stay in the intensive care unit (ICU). It is characterized by an acute onset of confusion, inattention, disorganized thinking, and altered levels of consciousness, often fluctuating in severity. Risk factors for ICU-associated delirium include advanced age, pre-existing cognitive impairment, severe illness, prolonged mechanical ventilation, and the use of sedative medications like benzodiazepines [1,2]. The condition not only prolongs hospital stays but is also linked to higher mortality rates, long-term cognitive decline, and increased healthcare costs. Early recognition through routine screening using tools like the Confusion Assessment Method for the ICU (CAM-ICU) is crucial for timely intervention [3,4].

Preventing and managing ICU delirium requires a multifaceted approach: minimizing sedation, maintaining sleep-wake cycles, and early mobilization. Non-pharmacological strategies like reorientation, daylight exposure, and family involvement can reduce delirium incidence [5]. If medications are needed, antipsychotics may be used cautiously, though evidence is limited. Treating underlying causes (e.g., infections, metabolic issues) is crucial. Prioritizing delirium prevention in ICU protocols improves both short-term recovery and long-term outcomes [6-10].

Ramelteon, a melatonin receptor agonist, has shown promise in reducing the incidence of delirium and potentially shortening ICU stays, particularly in high-risk patients (with comorbidities or risk factors of delirium) [11]. Unlike traditional sedatives, ramelteon mimics the natural sleep-wake cycle by selectively targeting MT1 and MT2 receptors in the brain, promoting sleep without significant respiratory depression or cognitive impairment. Studies suggest that ramelteon may help regulate circadian rhythms, which are often disrupted in critically ill patients, thereby lowering delirium risk [11-13]. These findings highlight its potential as a non-habit-forming alternative to conventional sedatives, which are known to exacerbate delirium.

The potential of ramelteon to reduce ICU stay duration is linked to its ability to mitigate delirium, a major contributor to prolonged hospitalization. By improving sleep quality and reducing delirium episodes, ramelteon may facilitate faster weaning from mechanical ventilation and earlier mobilization, both of which are associated with shorter ICU stays [11-13].

Existing evidence on ramelteon’s efficacy in reducing ICU delirium and shortening hospital stays is promising but limited, with studies showing mixed results due to the presence of confounders, particularly in ICU patients with other comorbidities. Given delirium’s impact on mortality, ICU length of stay, and healthcare costs, further randomized controlled trials are needed to confirm ramelteon’s role in delirium prevention, define its ideal use criteria, and assess its potential to improve both short- and long-term patient outcomes.

This study aimed to evaluate the efficacy and safety of ramelteon versus placebo in reducing ICU stay duration and delirium incidence while improving sleep quality in critically ill patients. The primary objective was to compare ICU length of stay between the two groups, while secondary objectives included assessing delirium occurrence and duration, nighttime awakenings, uninterrupted sleep nights, and mean sleep hours in non-intubated patients. We hypothesized that ramelteon would significantly shorten ICU stays, decrease delirium incidence rates, and enhance sleep metrics compared to placebo, potentially offering a non-sedative strategy for delirium prevention and ICU recovery optimization.

## Materials and methods

This study was an open-label, randomized controlled trial with a parallel 1:1 allocation ratio, conducted at the Department of Trauma and Emergency of a tertiary care hospital in Eastern India. The total study duration was six months following approval from the Institutional Ethics Committee (IEC). The trial was registered with the Clinical Trial Registry of India (CTRI) with registration number CTRI/2023/11/0256721.

Study population

The study included adult patients over 18 years of age, of either gender, who were admitted to the ICU during the study period and were able to receive medications orally or via a nasogastric tube within the first 48 hours of ICU admission. Patients were excluded if they were already receiving ramelteon or other drugs having inductive or inhibitory effects on delirium prior to ICU admission, had a known allergy to ramelteon, or were pregnant or lactating women.

Intervention

Eligible participants were randomly assigned using computer-generated random numbers at a 1:1 ratio to the ramelteon group (ramelteon: 8 mg/d) or the placebo group (placebo; 1 g/d of lactose powder) at the end of the baseline assessment.

Based on group allocation, patients received either ramelteon or a placebo at 20:00 hours each day until their discharge from the ICU. ICU nurses provided uniform care to all patients, which included routine preventive measures, minimizing unnecessary immobilization, and maintaining regular verbal interaction. Family visits were restricted to two sessions daily: between 11:00 a.m. and 12:00 noon and between 3:00 p.m. and 4:00 p.m. After 10:00 p.m., lighting and noise levels in the ICU were minimized to promote rest. At baseline, demographic details, medical history, and medication history were documented. Trained ICU nurses assessed patients using the Richmond Agitation-Sedation Scale (RASS) every four hours [14], except when patients were found to be asleep.

Outcomes

The primary outcome of the study was the duration of ICU stay. Secondary outcome included the incidence and duration of delirium during ICU stay and the clinical condition at discharge. Delirium was evaluated every four hours using the Confusion Assessment Method for the ICU (CAM-ICU) by trained ICU nurses, with assessments omitted if patients were asleep, and supplemented by evaluations from ICU physicians as needed [15].

For sleep-related outcomes, the number of nightly awakenings, the proportion of nights without awakenings relative to total ICU nights, and the average sleep duration in non-intubated patients were assessed. Outcome parameters were also assessed retrospectively through review of electronic medical records, including nursing notes and observational data.

Sample size

With anticipated 6 ± 1 days of average ICU stay, it was estimated that a sample size of 26 patients per group would be needed for detecting a reduction of the duration of ICU stay by one day (24 hours) in the ramelteon group, which we assumed as “a clinically significant effect size, with at least 95% statistical power and alpha value of 0.05.” So, we planned to recruit 30 patients in each group to cope with any expected attrition.

Statistical analysis

Statistical analysis of collected data was done through GraphPad version 8.4.3 (GraphPad Software, LLC, San Diego, CA). Continuous variables such as age, Acute Physiology and Chronic Health Evaluation II (APACHE II) score, Sequential Organ Failure Assessment (SOFA) score, ICU stay duration, delirium duration, and sleep parameters were analyzed using the unpaired t-test, assuming approximate normal distribution after applying the Shapiro-Wilk test. Categorical variables, including gender distribution, presence of dementia, mechanical ventilation status, and delirium frequency, were compared using Fisher’s exact test (if the chi-square test was not applicable). Differences in means were reported with standard error of the mean (SEM) and 95% confidence intervals (CI), and a p-value of less than 0.05 was considered statistically significant.

## Results

**Figure 1:**
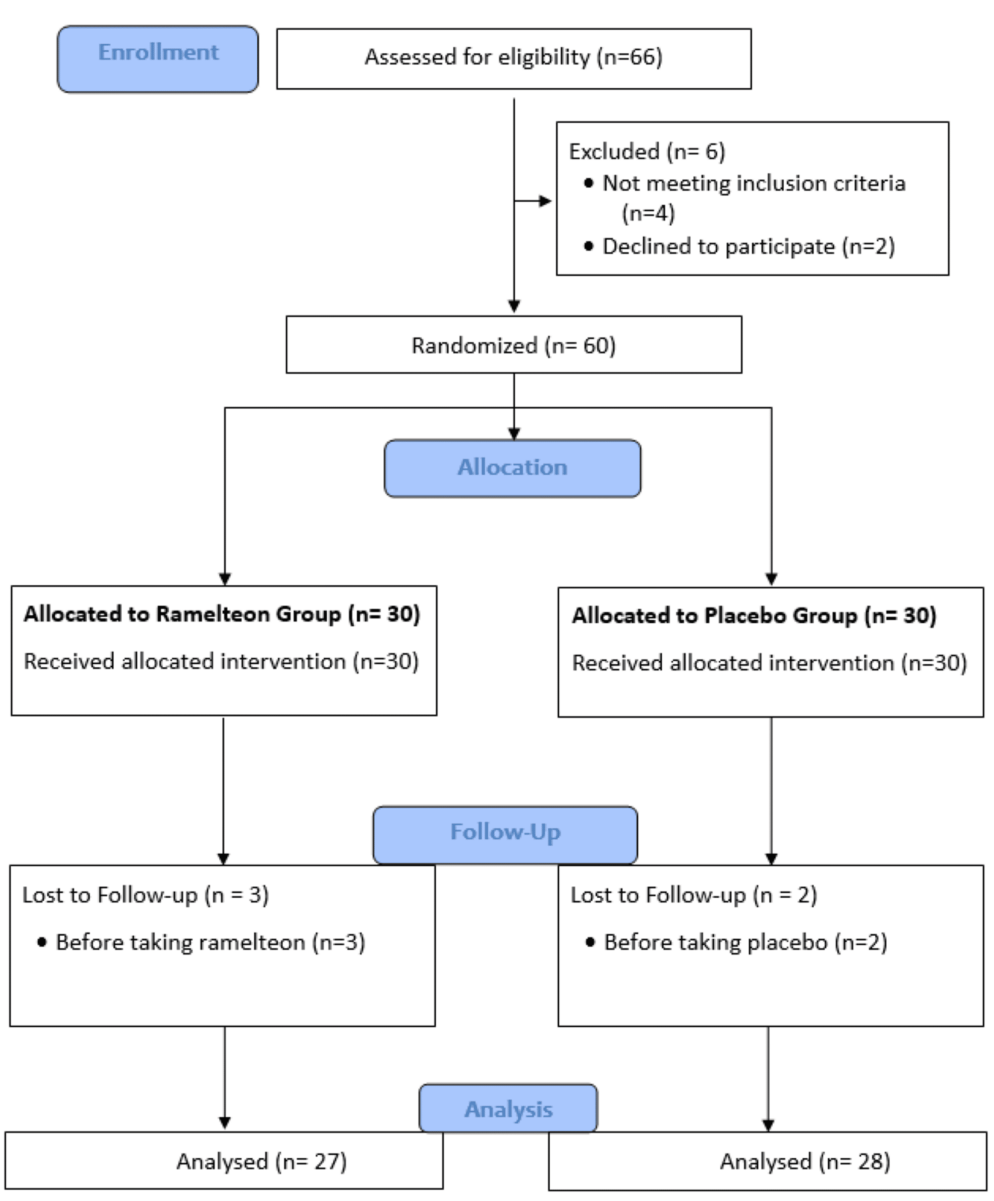
Consolidated Standards of Reporting Trials (CONSORT) 2010 flow diagram

Initially, 66 individuals were assessed for eligibility, of whom six were excluded (four for not meeting the inclusion criteria and two who declined participation), resulting in 60 participants being randomized. These were evenly allocated into two groups: 30 to the ramelteon group and 30 to the placebo group, with all receiving their assigned interventions. During follow-up, three participants in the ramelteon group and two in the placebo group were lost before taking the intervention. Ultimately, data from 27 participants in the ramelteon group and 28 in the placebo group were included in the final analysis (Figure 1). 

The baseline characteristics between the ramelteon and placebo groups were statistically comparable, indicating a well-balanced randomization. The mean age was similar (64.38 vs. 63.69 years, p = 0.7458), and the gender distribution showed no significant difference (19/27, 70.37% vs. 17/28, 60.71% male, p = 0.5731). The prevalence of dementia was slightly higher in the ramelteon group (4/27, 14.81% vs. 2/28, 7.14%), but this was not statistically significant (p = 0.4216). Both groups had comparable severity of illness, as reflected by similar APACHE II (23.87 vs. 24.06, p = 0.9299) and SOFA scores (7.98 vs. 8.37, p = 0.5698). The proportion of patients requiring mechanical ventilation was also nearly identical (11/27, 40.74% vs. 12/28, 42.86%, p > 0.9999) (Table 1).

**Table 1:** Comparison of baseline demographic and clinical characteristics between ramelteon and placebo groups *Unpaired t-test; **Fisher’s exact test; SD: standard deviation; APACHE: Acute Physiology and Chronic Health Evaluation; SOFA: Sequential Organ Failure Assessment

Variable	Ramelteon group (n = 27)	Placebo group (n = 28)	p-value
Age in years, mean ± SD	64.38 ± 7.39	63.69 ± 8.27	0.7458*
Male gender, n (%)	19 (70.37)	17 (60.71)	0.5731**
Dementia, n (%)	4 (14.81)	2 (7.14)	0.4216**
APACHE II score, mean ± SD	23.87 ± 7.41	24.06 ± 8.48	0.9299*
SOFA score, mean ± SD	7.98 ± 2.13	8.37 ± 2.86	0.5698*
Mechanical ventilation, n (%)	11 (40.74)	12 (42.86)	>0.9999**

Patients in the ramelteon group had a significantly shorter ICU stay compared to the placebo group, with a mean duration of 4.78 ± 1.59 days versus 6.23 ± 1.94 days, respectively. The difference in mean ICU stay was 1.75 days (± 0.4793 SEM), and this difference was statistically significant (p = 0.0006) (Table 2).

**Table 2:** Comparison of duration of ICU stay between ramelteon and placebo groups CI: confidence interval; SEM: standard error of the mean

	Ramelteon group	Placebo group
Number of patients (N)	27	28
Mean duration of ICU stay in days	4.78	6.23
Standard deviation (SD)	1.59	1.94
Difference in mean (P-R) ± SEM	1.450 ± 0.4793	
95% CI of difference	0.4887 to 2.411	
p-value (unpaired t test)	0.0038	

Although the incidence of delirium was lower in the ramelteon group (7/27, 25.93%) compared to the placebo group (13/28, 46.43%), this difference did not reach statistical significance (p = 0.1625). However, the duration of delirium was significantly shorter in the ramelteon group, with a mean of 18.83 hours versus 33.69 hours in the placebo group (p < 0.0001) (Table 3).

**Table 3:** Comparison of frequency and duration of delirium between ramelteon and placebo groups *unpaired t-test; **Fisher’s exact test; SD: standard deviation

Variable	Ramelteon group (n = 27)	Placebo group (n = 28)	p-value
Delirium frequency, n (%)	7 (25.93)	13 (46.43)	0.16254.47
Duration of delirium in hours, mean ± SD	18.83 ± 2.39	33.69 ± 4.51	<0.0001

Ramelteon was associated with improved sleep quality compared to placebo. Although the total sleep duration was slightly longer in the ramelteon group (7.38 vs. 6.87 hours), this difference was not statistically significant (p = 0.1886). However, patients receiving ramelteon experienced significantly fewer awakenings per night (0.79 vs. 1.28, p < 0.0001) and a higher proportion of nights without any awakening (0.56 vs. 0.35, p < 0.0001) (Table 4).

**Table 4:** Comparison of sleep variables between ramelteon and placebo groups SD: standard deviation

Variable	Ramelteon group (n = 27)	Placebo group (n = 28)	p-value (unpaired t-test)
Duration of sleep in hours, mean ± SD	7.38 ± 1.56	6.87 ± 1.27	0.1886
Number of awakenings per night, mean ± SD	0.79 ± 0.23	1.28 ± 0.26	<0.0001
Proportion of nights with no awakening, mean ± SD	0.56 ± 0.17	0.35 ± 0.09	<0.0001

## Discussion

Ramelteon, a selective melatonin receptor agonist, targets MT1 and MT2 receptors in the suprachiasmatic nucleus, which regulate the sleep-wake cycle. By promoting natural sleep architecture, ramelteon may help mitigate delirium risk and improve ICU outcomes. The present study evaluated the efficacy of ramelteon in reducing ICU stay, delirium incidence and duration, and improving sleep quality in critically ill patients, contributing to the growing body of research on melatoninergic interventions in critical care.

The study demonstrated several clinically significant effects of ramelteon. First, patients receiving ramelteon had a significantly shorter ICU stay (4.78 ± 1.59 days) compared to the placebo group (6.23 ± 1.94 days, p = 0.0006), suggesting a potential role in accelerating recovery or reducing complications. Second, while the frequency of delirium was lower in the ramelteon group (25.93% vs. 46.43%), this difference did not reach statistical significance (p = 0.1625). However, the duration of delirium was markedly shorter in the ramelteon group (18.83 vs. 33.69 hours, p < 0.0001), indicating a possible moderating effect on delirium severity. Third, ramelteon significantly improved sleep quality, with fewer nighttime awakenings (0.79 vs. 1.28, p < 0.0001) and a higher proportion of nights without awakenings (0.56 vs. 0.35, p < 0.0001). Total sleep duration, however, did not differ significantly between groups (p = 0.1886). These findings suggest that ramelteon may enhance sleep continuity without necessarily extending total sleep time, which could be particularly beneficial in the ICU environment.

The results of the present study align closely with those of Nishikimi et al., who also reported a trend toward reduced ICU stay and significantly lower delirium incidence with ramelteon [16]. Both studies noted improvements in sleep quality, reinforcing the consistency of ramelteon's effects on sleep architecture. However, the present study demonstrated stronger statistical significance for ICU stay reduction, possibly due to differences in patient populations or study design. In contrast, Jaiswal et al. and Oh et al. found no significant benefits of ramelteon in postoperative cardiac and orthopedic surgical patients, respectively [17,18]. This discrepancy suggests that ramelteon's efficacy may be context-dependent, with more pronounced effects in medical or mixed ICU populations compared to surgical cohorts, where factors like anesthesia and pain may dominate delirium risk.

Hatta et al. reported a striking reduction in delirium incidence (3% vs. 32%, p = 0.003) in elderly medical patients, supporting the preventive potential of ramelteon [19]. The present study's non-significant reduction in delirium frequency may reflect differences in delirium assessment timing or patient acuity. Romero et al., in a retrospective analysis, found no difference in delirium incidence between ramelteon, melatonin, and control groups, highlighting the variability in real-world effectiveness [20]. Despite these mixed results, Zhang et al.'s meta-analysis supports the overall benefits of melatoninergic agents, showing pooled reductions in delirium prevalence and ICU stay, consistent with the present study's findings [21].

The present study adds to the evidence that ramelteon may improve ICU outcomes by reducing delirium duration and shortening ICU stays, particularly in non-surgical populations. The significant improvement in sleep quality underscores its potential role in addressing sleep disruption, a modifiable risk factor for delirium. However, the variability in results across studies suggests that ramelteon's benefits may be most pronounced in specific patient subgroups, such as medical ICU patients or those with preexisting sleep disturbances.

This study has several limitations, including a small sample size. The single-center design and open-label nature of the trial could introduce bias, despite randomization. The exclusion of intubated patients from sleep assessments further restricts the applicability of the results to a broader ICU population. Finally, the short study duration (six months) and lack of long-term follow-up preclude conclusions about the sustained effects of ramelteon beyond the ICU stay.

Future research should focus on larger, multicenter trials to confirm these findings and identify optimal dosing regimens and patient populations. Additionally, studies should explore the interaction between ramelteon and other ICU interventions, such as sedation protocols and environmental modifications, to maximize its clinical impact.

## Conclusions

In conclusion, the present study supports the use of ramelteon in ICU settings to reduce delirium duration and ICU stay, with notable improvements in sleep quality. While its effects on delirium incidence remain inconsistent across studies, the cumulative evidence suggests that ramelteon is a promising prophylactic agent, particularly in medical or mixed ICU populations. Further research is needed to refine its clinical application and establish standardized protocols for its use in critical care.
